# Endoplasmic reticulum stress-related gene expression causes the progression of dilated cardiomyopathy by inducing apoptosis

**DOI:** 10.3389/fgene.2024.1366087

**Published:** 2024-04-18

**Authors:** Jinhao Chen, Xu Yang, Weiwen Li, Ying Lin, Run Lin, Xianzhen Cai, Baoxin Yan, Bin Xie, Jilin Li

**Affiliations:** ^1^ Department of Cardiology, Second Affiliated Hospital of Shantou University Medical College, Shantou, Guangdong, China; ^2^ Shantou University Medical College, Shantou, Guangdong, China

**Keywords:** dilated cardiomyopathy, endoplasmic reticulum stress, apoptosis, immune regulation, gene, bioinformatics

## Abstract

**Background:** Previous studies have shown that endoplasmic reticulum stress (ERS) -induced apoptosis is involved in the pathogenesis of dilated cardiomyopathy (DCM). However, the molecular mechanism involved has not been fully characterized.

**Results:** In total, eight genes were obtained at the intersection of 1,068 differentially expressed genes (DEGs) from differential expression analysis between DCM and healthy control (HC) samples, 320 module genes from weighted gene co-expression network analysis (WGCNA), and 2,009 endoplasmic reticulum stress (ERGs). These eight genes were found to be associated with immunity and angiogenesis. Four of these genes were related to apoptosis. The upregulation of MX1 may represent an autocompensatory response to DCM caused by a virus that inhibits viral RNA and DNA synthesis, while acting as an autoimmune antigen and inducing apoptosis. The upregulation of TESPA1 would lead to the dysfunction of calcium release from the endoplasmic reticulum. The upregulation of THBS4 would affect macrophage differentiation and apoptosis, consistent with inflammation and fibrosis of cardiomyocytes in DCM. The downregulation of MYH6 would lead to dysfunction of the sarcomere, further explaining cardiac remodeling in DCM. Moreover, the expression of genes affecting the immune micro-environment was significantly altered, including TGF-β family member. Analysis of the co-expression and competitive endogenous RNA (ceRNA) network identified XIST, which competitively binds seven target microRNAs (miRNAs) and regulates MX1 and THBS4 expression. Finally, bisphenol A and valproic acid were found to target MX1, MYH6, and THBS4.

**Conclusion:** We have identified four ERS-related genes (MX1, MYH6, TESPA1, and THBS4) that are dysregulated in DCM and related to apoptosis. This finding should help deepen understanding of the role of endoplasmic reticulum stress-induced apoptosis in the development of DCM.

## Graphical Abstract

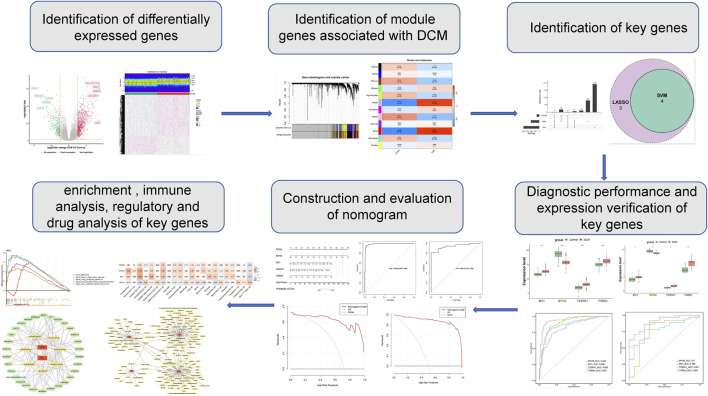

## Background

Dilated cardiomyopathy (DCM) is a common cause of systolic heart failure (HF) and is characterized by cardiac dilatation and low left ventricular ejection fraction (LVEF) (Li et al., 2023). The European Society of Cardiology (ESC) defines DCM as a disease that is characterized by left ventricular (LV) or biventricular dilatation and systolic dysfunction, in the absence of conditions causing abnormal loading (hypertension and valvular disease) or coronary artery disease severe enough to cause systolic abnormalities (Schultheiss et al., 2019). The etiology of DCM can be genetic, environmental, or the result of an interaction between genetic susceptibility and environmental factors (Kaski and Cannie, 2023). It has been shown that approximately 50% of patients with idiopathic DCM have familial genetic predisposition, and the disease is inherited in predominantly autosomal dominant fashion (Schultheiss et al., 2019). Alterations in the expression and function of some genes have been shown to impair cardiomyocyte function in a variety of ways, such as through titin splicing, by altering the threshold for the electrical excitation of sodium channels, by disrupting the assembly of nuclear fibrillar proteins, and by reducing transcriptional activity (Jordan et al., 2021). Therefore, screening for genetic biomarkers is important for the early prevention, diagnosis, and treatment of DCM.

The subcellular mechanisms underpinning DCM are currently unknown. The role of endoplasmic reticulum stress (ERS) in the pathogenesis and progression of DCM has been discussed for decades (Pietrafesa et al., 2023). A number of factors can disrupt normal endoplasmic reticulum (ER) function and cause ERS, including hypoxia, hyperglycemia, oxidative stress, lipotoxicity, and inflammation (Zhang et al., 2021; Harding et al., 2023). In addition, apoptosis plays a crucial role in myocardial injury, and cardiomyocyte homeostasis involves several cellular processes, including ERS and autophagy (Fang et al., 2021; Kong et al., 2022). ERS signaling has cytoprotective effects and exists to maintain cardiomyocyte homeostasis (Jiang et al., 2021). However, prolonged ERS leads to cardiomyocyte dysfunction and apoptosis, and the presence of autophagic vesicles in the left ventricle of patients with dilated myocardia has been reported (Sanchez-Esteban et al., 2021). It is clear that ERS-associated apoptosis is involved in the pathogenesis of DCM.

It has been shown that the reoxidation of protein disulfide isomerase (PDI) leads to the accumulation of ROS and a hyperoxidative state in the ER, which increases the aggregation of proteins and stimulates apoptosis (Wang et al., 2020). Furthermore, prolonged ERS leads to the activation of pathways that eliminate defective cells, and this occurs in DCM (Martinez-Amaro et al., 2023). It has also been shown that plasma from patients with ischemic dilated cardiomyopathy (ICM) is enriched in miR-16-5p and promotes ER stress-induced apoptosis in cardiomyocytes *in vitro* (Calderon-Dominguez et al., 2021). Moreover, the administration of astaxanthin (AST) ameliorates ethanol-induced DCM by inhibiting cardiac ERS and the resulting apoptosis, and this involves GRP78 (Wang et al., 2021). Finally, mutations in FBXO32 cause DCM via upregulation of ERS-associated apoptosis (Al-Yacoub et al., 2021). These findings indicate that ERS-induced apoptosis promotes the development of DCM. Therefore, the relationship between ERS and apoptosis in DCM requires further investigation, and may represent a therapeutic target for DCM.

To date, the specific mechanism of action between ERS and DCM is not very clear. Therefore, in the present study, we aimed to identify genes that are dysregulated in DCM and associated with ERS and apoptosis through bioinformatic analyses, and to further characterize the mechanism of action of these genes *in silico* to identify potentially useful biomarkers of DCM.

## Results

### Eight genes were found to be associated with immune function and angiogenesis

**FIGURE 1:**
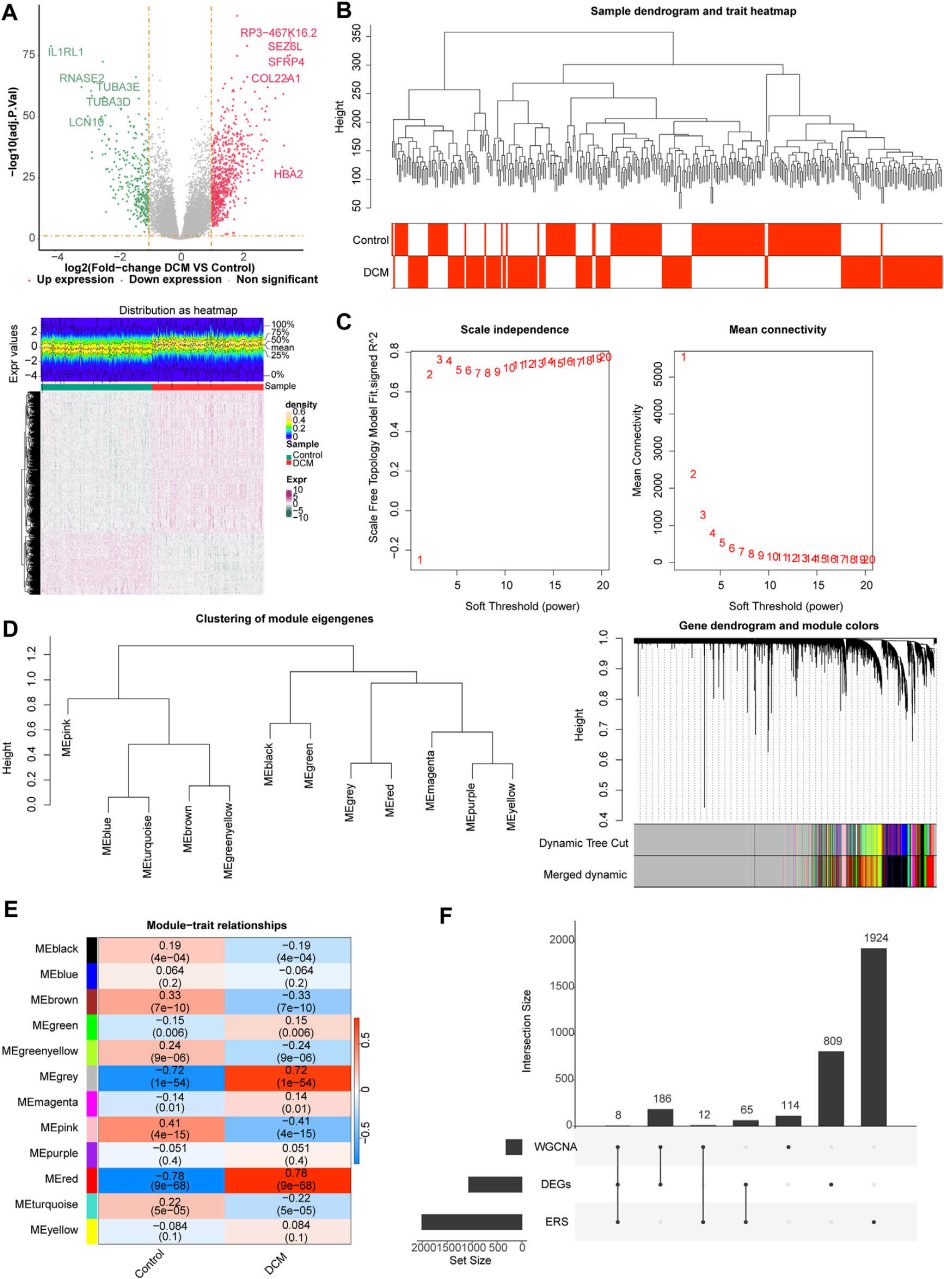
Differential expression analysis and weighted gene co-expression network analysis (WGCNA). **(A)** The volcano map and heat map of differentially expressed genes (DEGs) between dilated cardiomyopathy (DCM) and healthy control (HC) samples. **(B)** The samples in the GSE141910 dataset were clustered to remove the outlier. **(C)** Selection of the optimal soft-thresholding (power). **(D)** Hierarchical clustering of genes and module identification. **(E)** Heat map of the relationship between gene modules and clinical traits (DCM and HC). **(F)** Eight target genes obtained by overlapping DEGs, module genes, and endoplasmic reticulum stress (ERS)-related genes (ERGs).

One thousand sixty-eight differentially expression genes (DEGs, 748 upregulated and 320 downregulated) were identified using the 166 DCM and 166 healthy controls (HC) samples in the GSE141910 dataset (Figure 1A), and sample clustering analysis showed that there were no outlier samples (Figure 1B). When the optimal soft threshold value was identified to be 6, the network was close to scale-free distribution, and a total of 12 modules was obtained (Figures 1C, D. Correlation analysis showed that the MEred module had the strongest positive correlation with DCM (r = 0.78, *p* = 9e−68) (Figure 1E). Therefore, the MEred module was identified as the key module, and 320 genes in this module were identified as the module genes for subsequent analyses. A total of eight target genes (HLA-DQA1, MIR146A, MX1, MYH6, MYOC, TESPA1, THBS4, and TLR7) were obtained by studying the intersection of the 1,068 DEGs, 320 module genes, and 2,009 endoplasmic reticulum stress-related genes (ERGs) (Figure 1F).

**FIGURE 2:**
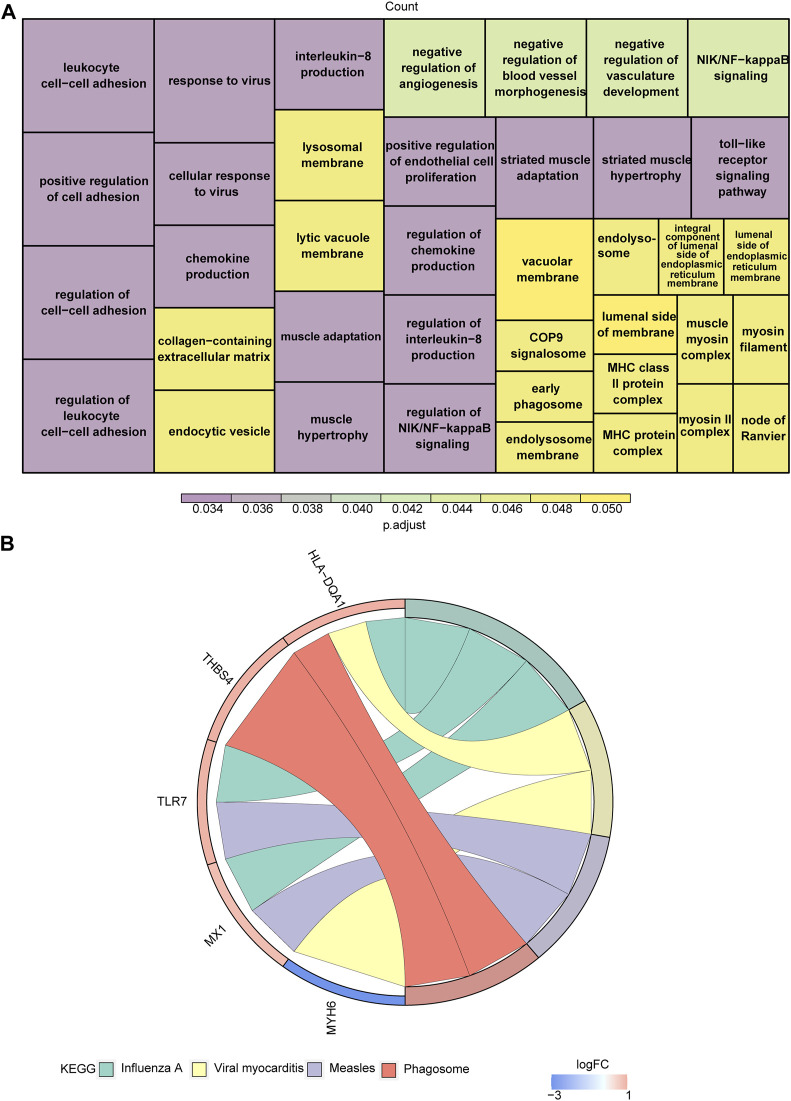
Functional enrichment analysis of target genes. **(A,B)** The Gene Ontology (GO) functions **(A)** and Kyoto Encyclopedia of Genes and Genomes (KEGG) pathways **(B)** enriched in target genes.

With respect to their function, these eight genes were found to be associated with striated muscle adaptation, the positive regulation of leukocyte cell-cell adhesion, the negative regulation of angiogenesis, the regulation of chemokine production, the regulation of interleukin-8 production, the regulation of NIK/NFκB signaling, the toll-like receptor signaling pathway, and 39 Gene Ontology (GO) functions (Figure 2A; Supplementary Table S2). In addition, these genes were associated with four Kyoto Encyclopedia of Genes and Genomes (KEGG) pathways, including influenza A, viral myocarditis, measles, and the phagosome (Figure 2B; Supplementary Table S3).

### MX1, MYH6, TESPA1, and THBS4 affect the progression of DCM

**FIGURE 3:**
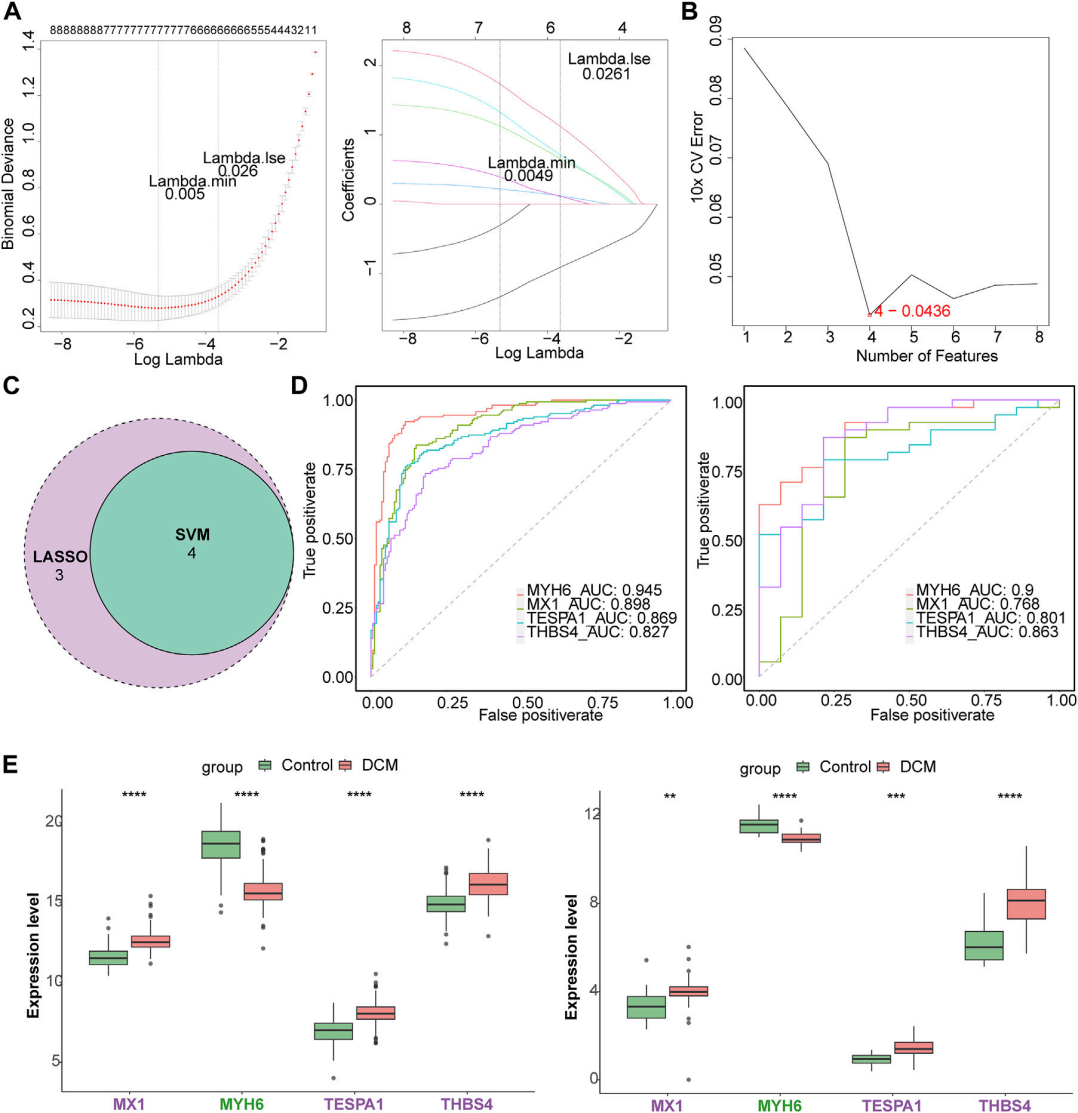
Identification of key genes. **(A)** The least absolute shrinkage and selection operator (LASSO) analysis of candidate genes. **(B)** Four genes were obtained by support vector machine recursive feature elimination (SVM-RFE) method. **(C)** The venn diagram of four candidate genes. **(D)** The receiver operating characteristic (ROC) curves of four candidate genes in the GSE141910 and GSE116250 datasets. **(E)** The expression of candidate genes in DCM and HC samples in the GSE141910 and GSE116250 datasets. **p < 0.01; ***p < 0.001; ****p < 0.0001.

Seven of the listed eight target genes (MIR146A, MX1, MYH6, MYOC, TESPA1, THBS4, and TLR7) were screened using least absolute shrinkage and selection operator (LASSO), and four (MX1, MYH6, TESPA1, and THBS4) were screened using support vector machine recursive feature elimination (SVM-RFE) (Figures 3A, B). Four candidate genes (MX1, MYH6, TESPA1, and THBS4) were obtained through examination of the intersection of these two sets of genes (Figure 3C). The area under the curves (AUCs) for all of these four candidate genes were >0.7 in both the GSE141910 and GSE116250 datasets (Figure 3D), and the trends in the expression of these genes were also consistent between the two datasets. MX1, TESPA1, and THBS4 were significantly upregulated, and MYH6 was significantly downregulated in the DCM samples in both the GSE141910 and GSE116250 datasets (*p* < 0.05) (Figure 3E). Therefore, all four of these genes were defined as genes of interest in DCM and were further studied.

**FIGURE 4:**
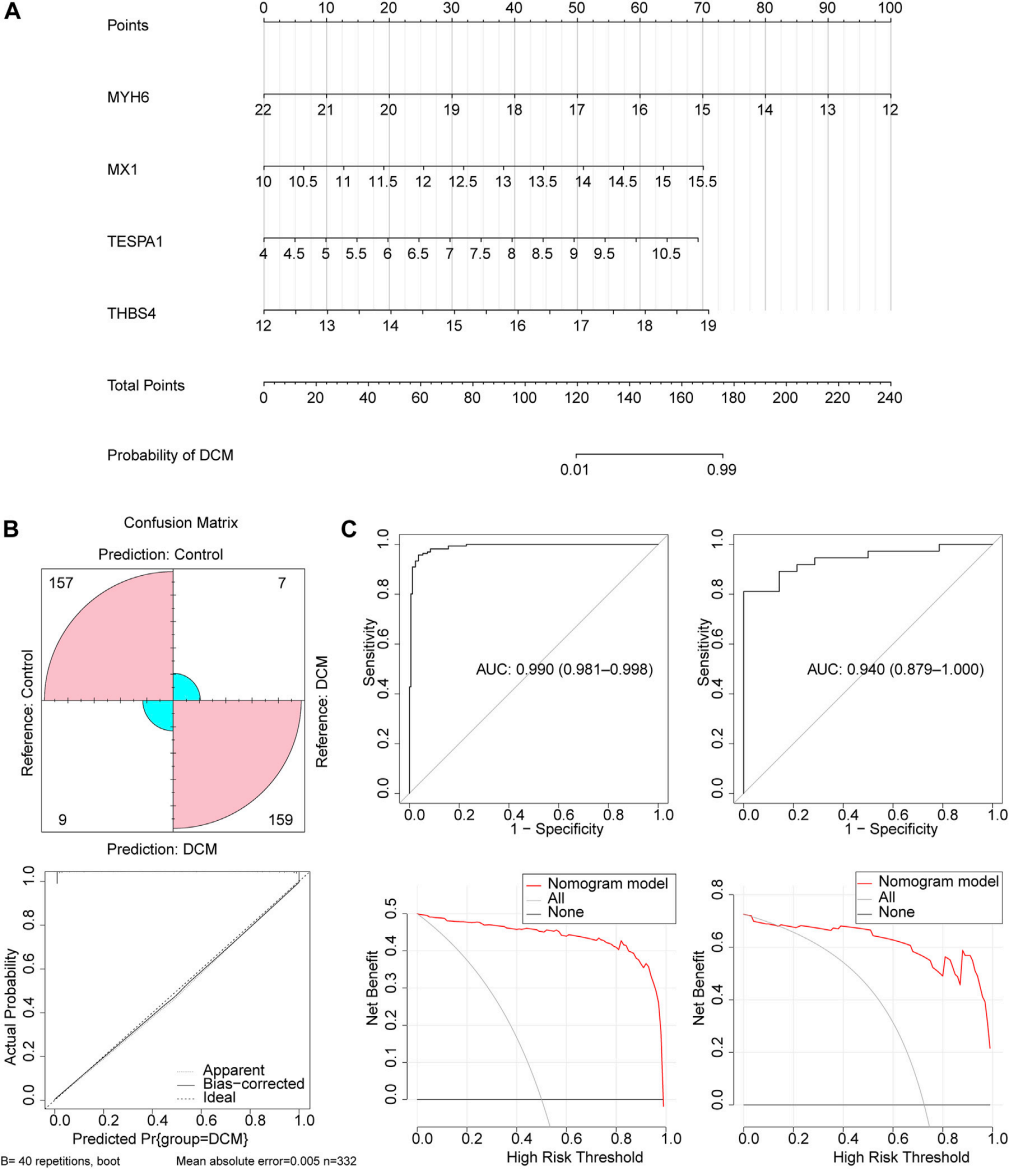
The construction and validation of the nomogram. **(A)** The nomogram created based on four key genes. **(B)** The calibration curve of the nomogram. **(C)** The ROC curve and decision curve of the nomogram in the GSE141910 and GSE116250 datasets.

Next, a nomogram comprising these four key genes was constructed (Figure 4A). The confusion matrix showed that most of the predictions were factual, and the calibration curve showed that the disease risk prediction was close to the truth (Figure 4B). In addition, the AUC for the nomogram was >0.8, and the decision curve showed that the benefit rate of the nomogram model was higher than that for each individual gene in both the GSE141910 and GSE116250 datasets (Figure 4C). Taken together, these findings indicate that this logistic regression model may represent a useful diagnostic model.

### The functions of the genes of interest are related to apoptosis

**FIGURE 5:**
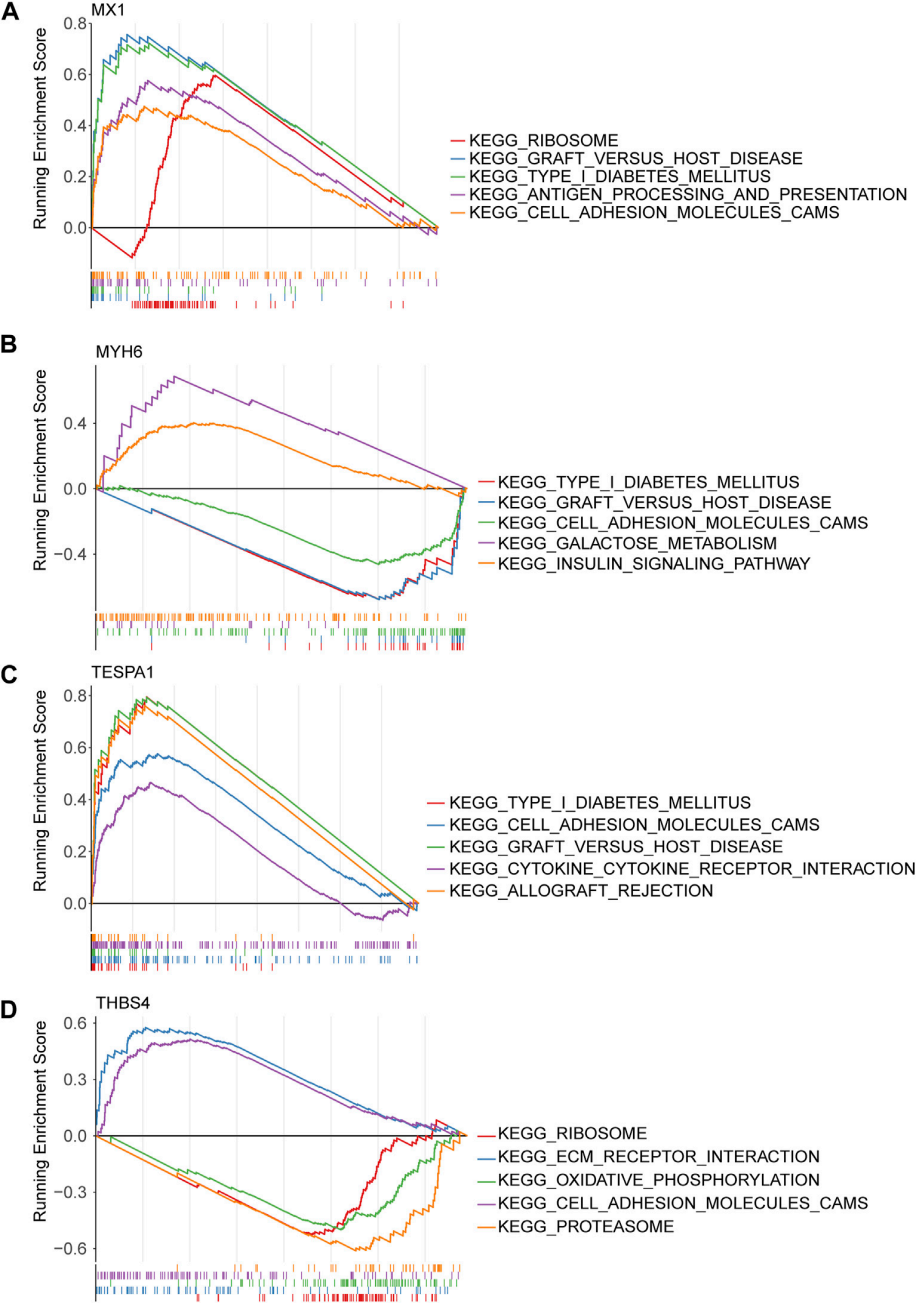
Gene set enrichment analysis (GSEA) of key genes. **(A)** MX1; **(B)** MYH6; **(C)** TESPA1; **(D)** THBS4.

Gene set enrichment analysis (GSEA) of the four genes of interest showed that they were associated with cytokine-cytokine receptor interactions, cell adhesion, apoptosis, MAPK, VEGF signaling pathways, and 22 KEGG pathways. MX1, TESPA1, and THBS4 were found to be associated with ECM receptor interaction, ERBB, the mTOR signaling pathway, and 18 KEGG pathways. In addition, THBS4 was found to be significantly negatively associated with oxidative phosphorylation, which was strongly associated with ERS (Figures 5A–D; Supplementary Tables S4–S7).

### The genes of interest play important roles in immunity

**FIGURE 6:**
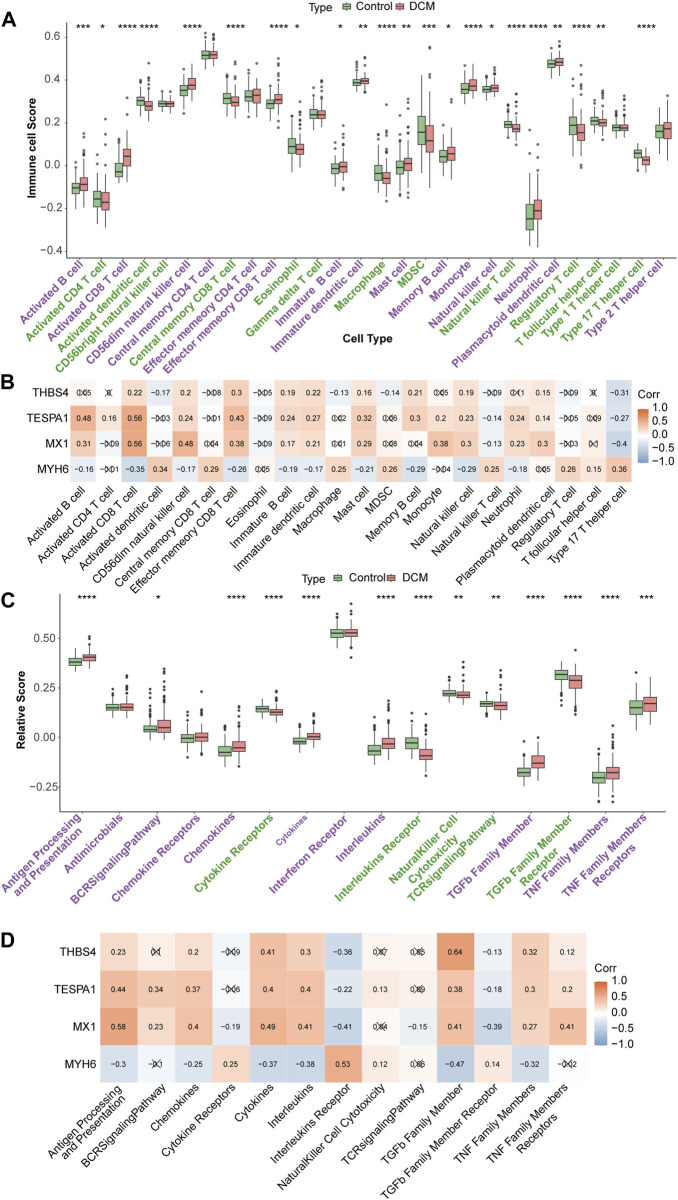
Immune infiltration analysis. **(A)** Discrepancies of immune cell scores in the DCM and HC samples. *p < 0.05; **p < 0.01; ***p < 0.001; ****p < 0.0001. **(B)** The relevance of key genes and differential immune cells. **(C)** The relative scores of immune reactions in the DCM and HC samples. *p < 0.05; **p < 0.01; ***p < 0.001; ****p < 0.0001. **(D)** The relevance of key genes and differential immune reactions.

Twelve types of immune cell were significantly overrepresented and 10 types were significantly underrepresented in the DCM group (*p* < 0.05). The expression of MX1, TESPA1, and THBS4 was significantly positively associated with the 12 upregulated immune cells and negatively associated with the 10 downregulated immune cells. In addition, the opposite relationships were identified for MYH6, which is consistent with the results of the expression and function analyses. The strongest negative correlation of MX1 was with the type 17 helper T cell population (r = −0.4), and its strongest positive correlation was with the activated CD8 T cell population (r = 0.56). The strongest positive correlation of TESPA1 was also with the activated CD8 T cell population (r = 0.56) (Figures 6A, B). Moreover, eight immune reactions were significantly upregulated and five were significantly downregulated in the DCM group (*p* < 0.05). The findings for MYH6 were opposite to those of the other genes of interest, which is consistent with the results of the expression and function analyses. The strongest negative correlation for MYH6 was with TGF-β family member (r = −0.47), whereas THBS4 showed the strongest positive correlation with TGF-β family member (r = 0.64) (Figures 6C, D).

### Potential regulatory mechanisms involving, and drugs potentially targeting, the genes of interest

**FIGURE 7:**
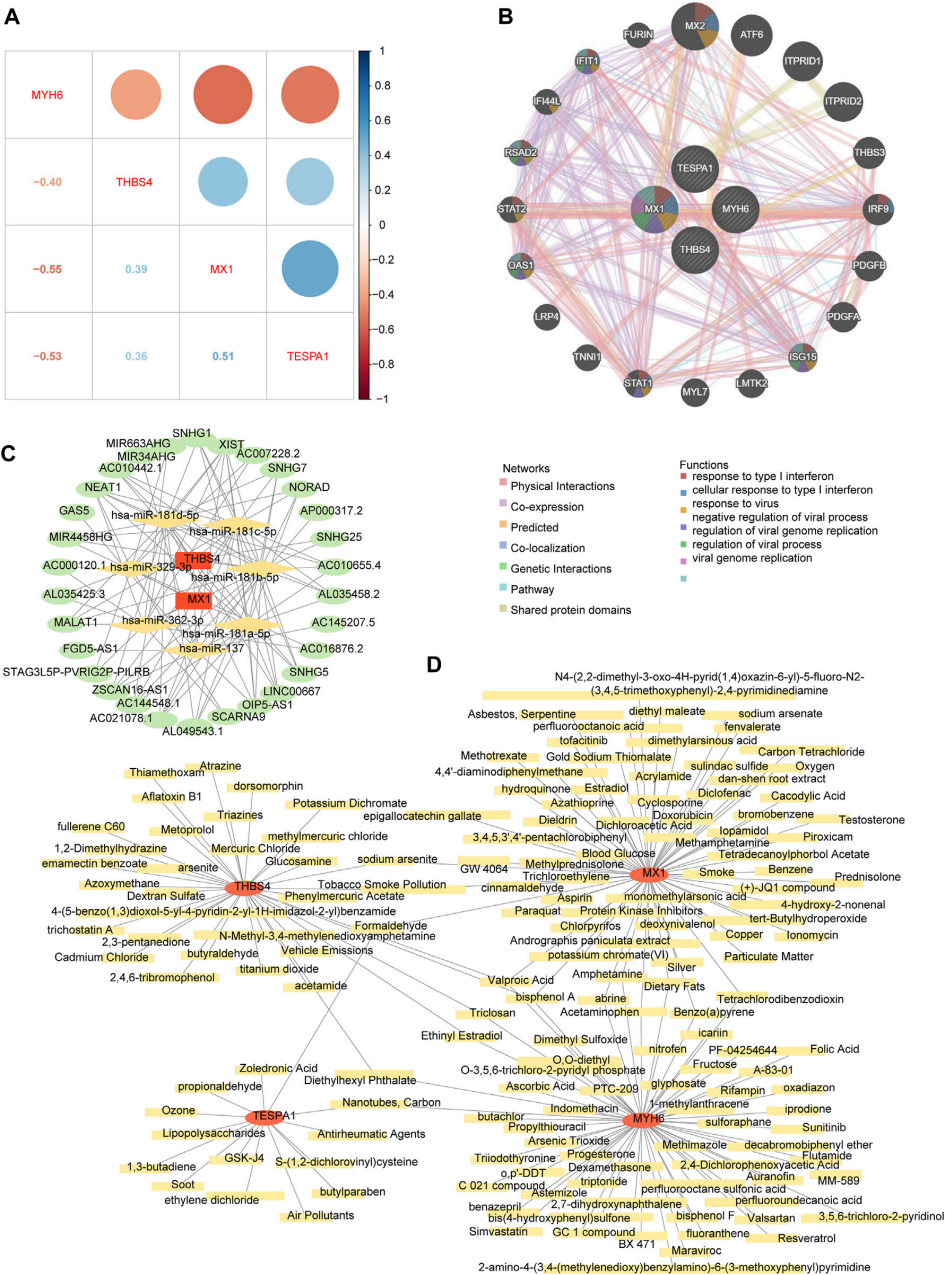
The potential regulatory mechanisms and drug prediction. **(A)** The relevance among four key genes. **(B)** The co-expression network of key genes. **(C)** The competitive endogenous RNA (ceRNA) network of key genes. The red graphic represents key genes, yellow represents microRNA (miRNA), and green represents long non-coding RNA (lncRNA). **(D)** The gene-drug network of key genes. The red graphic represents key genes and yellow represents targeted drugs.

The correlation analysis revealed positive correlations among MX1, TESPA1, and THBS4; whereas MYH6 showed negative correlations with the other three key genes. The strongest positive correlation was between MX1 and TESPA1 (r = 0.51), and the strongest negative correlation was between MX1 and MYH6 (r = −0.55) (Figure 7A). The co-expression network comprising the genes of interest and the 20 genes with the strongest correlations with the genes of interest showed that MX1 may be a hub gene, because it was found to be associated with the response to type I interferon and the regulation of viral genome replication. In addition, ISG15, OAS1, RSAD2, and IFIT1 were found to be associated with these functions (Figure 7B).

The competitive endogenous RNA (ceRNA) network regulatory network constructed using two genes of interest (MX1 and THBS4), seven microRNAs (miRNAs, containing hsa-miR-137, hsa-miR-181a-5p, hsa-miR-181b-5p, hsa-miR-181c-5p, hsa-miR-181d-5p, hsa-miR-329-3p, and hsa-miR-362-3p), and 30 long noncoding RNAs (lncRNAs) showed that XIST may competitively bind all seven miRNAs and regulate MX1 and THBS4. In addition, AC021078.1 and OIP5-AS1 may also competitively bind all five of the miRNAs targeting THBS4 (Figure 7C; Supplementary Table S8).

The chemical-gene network constructed using the four genes of interest and 159 chemicals showed that eight chemicals may target both MX1 and MYH6, three may target both MX1 and TESPA1, four may target MYH6 and THBS4, and three may target both MYH6 and THBS4. Specifically, bisphenol A and valproic acid were predicted to target all of MX1, MYH6, and THBS4 (Figure 7D; Supplementary Table S9).

## Discussion

In the present study, we have identified eight genes that are associated with inflammation and angiogenesis, and ERS is known to influence disease progression by regulating and activating angiogenesis (Zou et al., 2019; Hardy et al., 2021). Many previous studies have shown that macrophages differentiated from immune-infiltrating monocytes regulate myocardial inflammation and cardiac injury, and thereby accelerate abnormal ventricular remodeling and arterial vasculogenesis (Wong et al., 2021). The results of KEGG analysis suggested these genes principally play roles in pathways related to viral infection (influenza A), myocarditis (viral myocarditis), and the autophagosome (phagosome). It has been previously shown that damage caused by, and the immune response following, viral infection leads to myocarditis, and that cellular autophagy is involved in viral clearance and replication, which can lead to DCM (Tschöpe et al., 2021). The cellular fractions that were enriched on GO analysis were principally endocytic vesicles and lysosomal membranes (endocytic vesicles), organelles that are involved in cytophagy and autophagy and are functionally and structurally related to the fusion of vesicles with the endoplasmic reticulum (Nielsen et al., 2018; Li et al., 2020), further suggesting that cellular autophagy plays a role in DCM.

Four genes of interest, MYH6, MX1, TESPA1, and THBS4, were further studied by constructing machine learning models using these as hub genes. However, functional enrichment analysis showed that they are all related to apoptosis, which plays an important role in DCM. It is also worth noting that these four genes are related to the MAPK and VEGF signaling pathways; and that MX1, TESPA1, and THBS4 are also related to the ERBB and mTOR signaling pathways. The trends in the expression of the four hub genes were consistent and significant in both the training and validation sets, except for MYH6, which was expressed at lower levels in the DCM group, whereas the expression of the other three genes was higher in the DCM group than in the control group.

MYH6 encodes myosin heavy chain, an intracellular protein expressed in cardiac myocytes that has been identified as a major antigen in cardiac autoimmunity (Nielsen et al., 2018; Li et al., 2020). Its downregulation would lead to sarcomere dysfunction and, in turn, cardiac remodeling. In addition, there is evidence that MYH6 regulation of a miRNA-FOXO3 axis plays a key role in preventing myocardial apoptosis and excessive autophagy during the pathogenesis of DCM (Cheng et al., 2023). It has been reported that the mutation of MYH6 can lead to arrhythmia and sudden cardiac death in DCM(Zhao et al., 2021).

The MX1 gene encodes a guanosine triphosphate (GTP) metabolic protein that is involved in the cellular antiviral response. Expression of the encoded protein is induced by type I and type II interferons and it can inhibit the replication of several RNA and DNA viruses (Chen et al., 2021). High expression of this protein may inhibit viral replication in DCM-affected cells. It has been reported that MxA is an IFN-induced and MX1-expressed antiviral protein that localizes to and reorganizes cell membranes in association with ER, in a similar way to members of the large GTPase family that are constitutionally expressed (Davis et al., 2019).

TESPA1 is a protein-bridging subunit that binds and activates ip3r1 to control the release of Ca^2+^ from the ER (Parys and Vervliet, 2020), which is the main site of intracellular Ca^2+^ storage. When exogenous compounds enter cells, abnormal Ca^2+^ metabolism in the ER leads to ERS and thus apoptosis, which has been reported to be a feature of DCM and other heart diseases (Gao et al., 2022; Martinez-Amaro et al., 2023).The protein encoded by the THBS4 gene belongs to the platelet-responsive protein family. Members of the platelet-responsive protein family are adhesion glycoproteins that mediate intercellular and intercellular matrix interactions. The product of this gene can induce inflammation and fibrosis in cardiomyocytes by affecting macrophage differentiation and apoptosis (Rahman et al., 2020). In summary, the four genes of interest are involved in ERS, cell autophagy, and apoptosis in DCM, which might have important implications for the pathogenesis of DCM.

Analysis of the infiltrating immune cells revealed significantly different immune microenvironments in the control and DCM groups, with the four hubs being significantly associated with activated B cells, activated CD8 T cells, memory B cells, and natural killer cells; and all of these immune cell types were upregulated in DCM samples. Immune response analysis revealed the upregulation of antigen processing and presentation proteins, anti-microbial factors, B-cell receptor signaling, chemokines and cytokines, interferons, transforming growth factor-β, and tumor necrosis factor. CD8 T lymphocytes are the principal cellular component of the adaptive immune system, and during bacterial infection, B cells promote the activation of CD8 T cells to enhance antigen processing and presentation and B cell receptor signaling to resist infection. In contrast, natural killer cells are characterized by their non-specific effects to kill tumor cells and virally infected cells.

In DCM, viral replication leads to myocyte necrosis, and the MX1 activates the host’s innate immune system by presenting viral antigens (Kawada et al., 2021), leading to the proliferation and activation of a large number of immune cells, which produce chemokines and cytokines, such as TGF-β family member, which are involved in the recognition and killing of infected cells. During this process, viral damage causes the release of intracellular or surface antigens, which may become autoimmune targets, thus causing autoimmune tissue damage and cardiac inflammation (Lasrado and Reddy, 2020). Following tissue injury, monocytes and macrophages undergo significant phenotypic and functional changes in tissues. During this period, TGF-β, a key regulator of tissue repair and regenerative fibrosis, the principal function of which is to regulate the inflammatory process, plays an important role in the cardiac remodeling of injured cardiomyocytes and the pathogenesis of fibrosis (Frangogiannis, 2022). In addition, the Ang II-ATR1 signaling pathway, downstream of TGF-β, plays a key role in the post-inflammatory fibrotic remodeling that is involved in the development of DCM (Czepiel et al., 2022). Moreover, TGF-β can induce DCM by promoting ripk1-dependent apoptosis (Yin et al., 2022). These published findings are consistent with the upregulation of MX1 identified in the present analysis and the downregulation of MYH6, along with the negative correlation of the expression of MYH6 with TGF-β expression. Accordingly, we speculate that MX1 and MYH6, two ERS-related genes, mediate inflammatory responses that also involve TGF-β, thereby inducing cardiomyocyte apoptosis, which leads to the development of DCM.

We performed a correlation analysis of the genes of interest, and identified positive correlations between MX1, TESPA1, and THBS4; and negative correlations between MYH6 and the other three genes, consistent with the results of the expression and functional analyses. MX1 induces the production of interferon by presenting viral antigens, which reduces the expression of endogenous antigens, such as MYH6, and activates TESPA1 and THBS4, thereby activating specific immune pathways and promoting apoptosis.

Next, we constructed a ceRNA regulatory network to better understand the relevance of the lncRNAs, miRNAs, and regulatory hub genes. Two lncRNAs in this network, NEAT1 and XIST, can bind to multiple miRNAs and thereby regulate the expression of MX1 and THBS4. NEAT1 principally regulates subnuclear paraspeckles in nuclei, and paraspeckles and their family members can play a role in several biological processes, including cell differentiation, the response to viral infection, and oxidative stress (Guo et al., 2022). It has been shown that XIST is associated with a variety of cardiac diseases. XIST regulates mRNA expression through miRNA, which in turn affects the progression of cardiomyocyte hypertrophy (Chen et al., 2018; Xiao et al., 2019). In addition, XIST promotes the development of myocardial infarction through a similar approach (Zhou et al., 2019; Lin et al., 2020). Moreover, in this regulatory network, lncRNA FGD5-AS1 has been reported to be a key lncRNA in DCM (Schiano et al., 2021). FGD5-AS1 is an important regulatory gene in the heart and is involved in cell proliferation, migration, invasion, and the inhibition of apoptosis.

Other lncRNAs also play roles in important biological pathways. For example, by regulating mRNA expression through miRNA, AC145207.5 participates in cellular immunity, MIR34AHG is an ERS-related lncRNA (Chen et al., 2022a), SPAG9 activates the JNK signaling pathway (Liu et al., 2021a), and nucleolar RNA host genes all play roles in cardiac-related diseases. These regulated pathways are very similar to the biological processes that are dysregulated in DCM. In summary, XIST and SNHG5 may affect the activation of important pathways, such as the TGF-β and cAMP signaling pathways, in DCM by regulating multiple miRNAs, which in turn affect cellular fibrosis and apoptosis. Nucleolar RNA genes may also affect the function of vascular cells and cardiomyocytes through hsa-miR-181a-5p and hsa-miR-181b-5p, and MIR34AHG regulates ER function and thereby other cellular functions.

We have previously shown that bisphenol A is closely associated with various cardiovascular diseases, including myocardial infarction, arrhythmia, DCM, atherosclerosis, and hypertension (Zhang et al., 2020); and that the circulating triiodothyronine concentration is also associated with the prognosis of patients with DCM (Zhao et al., 2020). Valproic acid is a deacetylation inhibitor that protects the cardiac function of patients who have experienced myocardial infarction through the Foxm1 pathway, and therefore may also represent a potential therapeutic agent for DCM (Tian et al., 2019).

In summary, we have shown that four hub genes (MYH6, MX1, TESPA1, and THBS4) are associated with ERS-induced DCM and apoptosis. They may be involved in the etiology of DCM through effects on the MAPK, VEGF, ERBB, and mTOR signaling pathways. Furthermore, we identified associations of these four genes with the immunological phenotype of DCM, including with the size of the population of activated CD8 T cells, interleukin receptors, and antigen processing and presentation. Notably, MX1 and MYH6 may be associated with inflammatory responses and TGF-β activation, thereby inducing apoptosis, which is an important part of the pathogenesis of DCM. In addition, we have identified seven miRNAs and 30 lncRNAs that may be involved, including FGD5-AS1, which is involved in cell proliferation, migration, invasion, and the inhibition of apoptosis. Finally, we have identified 159 compounds that may represent potential therapeutic options. Of these, bisphenol A, triiodothyronine, and valproic acid may be useful for early interventions in DCM. However, experimental verification of the gene expression findings in animal models is required prior to any clinical assessment. In the future, we will further verify the function of key genes through animal model experiments.

## Materials and methods

### Data extraction

Two DCM-related transcriptome datasets were downloaded from the Gene Expression Omnibus (GEO) database. The GSE141910 dataset comprises data obtained from 166 patients with DCM and 166 HCs, and the GSE116250 dataset comprises data obtained from 37 patients with DCM and 14 HCs. All of the samples were LV tissue samples. In addition, a total of 2,009 ERGs were obtained from the GeneCards database (Relevance score >5) (version 5.14) (Supplementary Table S1).

### Functional enrichment analysis of DCM-related genes

The DEGs between the DCM and HC samples in the GSE141910 dataset were compared using the “limma” R package (Ritchie et al., 2015) (version 3.52.4) (|log_2_FC| ≥ 1, adjusted *p* < 0.05), then a co-expression network was constructed using “WGCNA” R package (Langfelder and Horvath, 2008) (version 1.71) to screen relevant module genes that were associated with DCM. Genes of interest were obtained by identifying the intersection among the DEGs, module genes, and ERGs using “ggvenn” (Yan, 2023) (version 0.1.9). Finally, functional enrichment analysis of these genes was conducted using “clusterProfiler” R package (Wu et al., 2021) (version 4.4.4) (adjusted *p* < 0.05).

### Identification of genes of interest and construction of a diagnostic model for DCM

The LASSO analysis using the glmnet package (Friedman et al., 2010) (version 4.14-4) and the SVM-RFE method using the e1071 package (version 1.7-11) (Jiang et al., 2020) were used to screen the selected genes, then candidate genes were obtained by comparing these two sets of genes. Next, receiver operating characteristic (ROC) curves for each candidate gene were constructed to study their ability to identify patients with DCM in both GSE141910 and GSE116250 datasets, using the “pROC” R package (Robin et al., 2011) (version 1.18.0). In addition, the expression of these genes in DCM and HC samples in both the GSE141910 and GSE116250 datasets was compared. Genes with areas under the ROC curve (AUCs) > 0.7 and with consistent expression trends in both the GSE141910 and GSE116250 datasets were regarded as genes of interest in DCM.

A diagnostic model (logistic regression model, nomogram) was then constructed using these genes using the “rms” R package (version 6.3-0) (Liu et al., 2021b); a confusion matrix was constructed; and a calibration curve, ROC curve, and decision curve were drawn to determine the validity of the diagnostic model.

### Functional analysis of the genes of interest

Pearson correlation coefficients for the relationships between each gene of interest and with all the expressed genes were calculated, and GSEA was performed to study the pathways associated with each gene using “clusterProfiler” R package (Wu et al., 2021) (adjusted *p* < 0.05).

### Analysis of the immune micro-environment

To explore the role of the genes of interest in the immune micro-environment, the proportions of 28 immune cells and 17 immune reactions were calculated for the DCM and HC samples using the “ssGSEA” algorithm and compared using the Wilcoxon signed-rank test. Moreover, the relationships between the genes of interest and the immune cell populations, and between these genes and the differentially activated immune reactions, were further studied using Spearman’s correlation.

### Analysis of the potential regulatory mechanisms involving the genes of interest

Pearson correlations for the relationships among the genes of interest were calculated, then a co-expression network comprising the genes of interest and the 20 genes with the closest correlations with these genes was constructed using the GeneMANIA online tool.

Next, the miRNAs targeting the genes of interest were predicted using the Starbase and TargetScan databases (Chen et al., 2022b), and miRNAs of interest were identified by studying the intersection of these two groups of predicted miRNAs. The lncRNAs targeting these miRNAs were also predicted using the Starbase and TargetScan databases (clipExpNum ≥5), and lncRNAs of interest were obtained by studying the intersection of these two groups of predicted lncRNAs. Finally, a competitive endogenous RNA (ceRNA) network was constructed using “Cytoscape” (Su et al., 2014) (version 3.9.1).

### Prediction of drugs targeting the genes of interest

We also predicted the drugs that would target the genes of interest using the Comparative Toxicogenomics database, (https://ctdbase.org/), and a chemicals-gene network was constructed using “Cytoscape” (Su et al., 2014) (version 3.9.1).

### Statistical analysis

All analyses were conducted using R (R Foundation for Statistical Computing, Vienna, Austria). Differences between two groups were identified using the Wilcoxon signed-rank test. If not specified otherwise above, *p* < 0.05 was regarded as indicating statistical significance.

## Data Availability

The datasets presented in this study can be found in online repositories. The names of the repository/repositories and accession number(s) can be found in the article/Supplementary Material.

## Supplementary material

SUPPLEMENTARY TABLE S1 The list of 2,009 ERS-related genes (ERGs) obtained from the GeneCards database.

SUPPLEMENTARY TABLE S2 The GO functions enriched in eight target genes.

SUPPLEMENTARY TABLE S3 The KEGG pathways enriched in eight target genes.

SUPPLEMENTARY TABLE S4 The GSEA enrichment analysis of MX1.

SUPPLEMENTARY TABLE S5 The GSEA enrichment analysis of MYH6.

SUPPLEMENTARY TABLE S6 The GSEA enrichment analysis of TESPA1.

SUPPLEMENTARY TABLE S7 The GSEA enrichment analysis of THBS4.

SUPPLEMENTARY TABLE S8 The genes, miRNAs, and lncRNAs in the ceRNA network.

SUPPLEMENTARY TABLE S9 The list of drugs targeting key genes.

## Abbreviations

ERS, endoplasmic reticulum stress; DCM, dilated cardiomyopathy; GEO, Gene Expression Omnibus; HC, healthy control; ERG, ERS-related gene; DEGs, differentially expressed genes; WGCNA, weighted gene co-expression network analysis; LASSO, least absolute shrinkage and selection operator; SVM-RFE, support vector machine recursive feature elimination; ROC, receiver operating characteristic; AUC, area under the curve; GSEA, gene set enrichment analysis; miRNA, microRNA; lncRNA, long non-coding RNA; ceRNA, competitive endogenous RNA; GO, Gene Ontology; KEGG, Kyoto Encyclopedia of Genes and Genomes.
